# Exploring Transcriptional Regulation of Beta Cell SASP by Brd4-Associated Proteins and Cell Cycle Control Protein p21

**DOI:** 10.3390/epigenomes8010010

**Published:** 2024-03-06

**Authors:** Jasmine Manji, Jasmine Pipella, Gabriel Brawerman, Peter J. Thompson

**Affiliations:** 1 Diabetes Research Envisioned and Accomplished in Manitoba (DREAM) Theme, Children’s Hospital Research Institute of Manitoba, Winnipeg, MB R3E 3P4, Canada; 2 Department of Physiology & Pathophysiology, Rady Faculty of Health Sciences, University of Manitoba, Winnipeg, MB R3E 3P4, Canada

**Keywords:** pancreatic beta cells, cellular senescence, type 1 diabetes, bromodomain extra-terminal domain proteins, SASP

## Abstract

Type 1 diabetes (T1D) is a metabolic disease resulting from progressive autoimmune destruction of insulin-producing pancreatic beta cells. Although the majority of beta cells are lost in T1D, a small subset undergoes senescence, a stress response involving growth arrest, DNA damage response, and activation of a senescence-associated secretory phenotype (SASP). SASP in beta cells of the nonobese diabetic (NOD) mouse model of T1D and primary human islets is regulated at the level of transcription by bromodomain extra-terminal (BET) proteins, but the mechanisms remain unclear. To explore how SASP is transcriptionally regulated in beta cells, we used the NOD beta cell line NIT-1 to model beta cell SASP and identified binding partners of BET protein Brd4 and explored the role of the cyclin-dependent kinase inhibitor p21. Brd4 interacted with a variety of proteins in senescent NIT-1 cells including subunits of the Ino80 chromatin remodeling complex, which was expressed in beta cells during T1D progression in NOD mice and in human beta cells of control, autoantibody-positive, and T1D donors as determined from single-cell RNA-seq data. RNAi knockdown of p21 during senescence in NIT-1 cells did not significantly impact viability or SASP. Taken together, these results suggest that Brd4 interacts with several protein partners during senescence in NIT-1 cells, some of which may play roles in SASP gene activation and that p21 is dispensable for the SASP in this beta cell model.

## 1. Introduction

Type 1 diabetes (T1D) is a chronic metabolic disorder that results from progressive autoimmune destruction of insulin-producing pancreatic beta cells in the islets of Langerhans. T1D mainly affects children and young people between ages 5–18 but can occur at any age, with an estimated prevalence of T1D being around 1 in 1000 and increasing in incidence at 3–4% per year over the past 16 years in the U.S. [1]. While a major focus of the field has been the study of immune system dysfunction in T1D, recent years have seen a growing interest in understanding the role of stress and dysfunction in islets/beta cells in promoting disease progression. Established programs of beta cell stress during T1D include the unfolded protein response (UPR) and type I interferon response [2]. Preclinical studies on these stress pathways support their involvement in the pathogenesis and onset of symptomatic diabetes in the gold standard T1D mouse model, the nonobese diabetic (NOD) mouse [3,4]. Recent phase II clinical trials have shown promising results in slowing beta cell decline after diagnosis using agents to modulate these pathways [5,6,7]. Thus, beta cell stress pathways have emerged as important therapeutic targets for T1D and a better understanding of their molecular mechanisms will improve efforts to develop new treatments.

We found previously that a subset of beta cells (around 5–20%) exhibits features of a classical senescence stress response during the development of T1D in NOD mice and in humans [8]. Cellular senescence is a stress response program defined by several features, such as cell cycle/growth arrest, DNA damage response, resistance to intrinsic apoptosis and a unique immunogenic secretome termed the senescence-associated secretory phenotype (SASP) [9]. SASP contains a variety of chemokines, cytokines, proteases, shed receptors, and other molecules that promote immune surveillance and senescent cell removal [10]. However, senescent beta cells with SASP accumulate before and during the development of diabetes in NOD mice. Notably, eliminating senescent beta cells in NOD mice using Bcl-2 family inhibitor senolytic compounds significantly delays diabetes in this model [8], indicating that senescent beta cells accelerate disease progression.

SASP is activated by epigenetic mechanisms, including bromodomain extra-terminal (BET) domain proteins in NOD mouse islets and human islets [11]. The BET protein family includes bromodomain-containing proteins 2, 3, and 4 (Brd2, Brd3, Brd4) in somatic cells, which are expressed broadly in most cell types [12]. BET proteins are recruited to DNA by transcription factors and bind acetylated histone tails to activate gene expression from promoters or enhancers [12]. Consistent with its role in activating SASP during senescence in other cell types [13], we found that Brd4 was bound to SASP gene promoters and enhancers in NOD mouse islets [11]. Small molecule inhibition of Brd4 chromatin binding with iBET-762 [14] reduced SASP gene expression and protein secretion. iBET-762 also diminished SASP from human islets induced to senescence by DNA damage ex vivo [11]. Remarkably, BET protein inhibition with iBET-762 or an earlier generation inhibitor iBET-151 delayed diabetes in NOD mice [11,15], and we linked this effect with a reduction of SASP in beta cells in NOD mice [11].

Although BET proteins are required for SASP gene activation in beta cells during the development of T1D, the mechanisms remain unclear, and it is not known what recruits Brd4 to SASP gene regulatory regions in beta cells. While it is recognized that the most suitable model to study this would be T1D patient primary islets, these donor specimens are very depleted of beta cells and are extremely difficult to obtain through tissue procurement networks. Therefore, we utilized beta cell line and islet culture models. Although cell culture systems are less physiologically relevant, nevertheless, they still permit basic molecular mechanisms to be investigated. We established previously a mouse beta cell line model in which to study senescence phenotypes on the relevant genetic background to T1D using the NIT-1 beta cell line [16], derived from a 10 week old prediabetic female NOD mouse [17]. NIT-1 beta cells respond similarly to primary NOD mouse islet cells to inflammatory stimuli such as cytokines [17] and have been used extensively in the field to model molecular mechanisms of beta cell stress in T1D. Although these cells are transformed with Simian Virus 40 large T antigen, following sub-lethal DNA double-strand break damage, they undergo a growth arrest, activate a canonical DNA damage response, and stably upregulate p21 [16], recapitulating features of senescent primary beta cells in NOD mice [8] and human islet beta cells in a DNA damage-induced senescence model [18]. Senescent NIT-1 cells also produce a SASP involving insulin-like growth factor binding protein 3 (Igfbp3) and plasminogen activator inhibitor (Pai1) [16], two factors in common with the SASP of primary NOD beta cells [8]. These observations suggest that NIT-1 cells could be a useful cell line model in which to study mechanisms controlling SASP in beta cells.

Since BET proteins frequently interact with a wide range of different nuclear proteins that could regulate their activity and recruitment to SASP genes, we investigated the mechanism of SASP gene activation by identifying binding partners of Brd4 during senescence in NIT-1 cells and performing a siRNA knockdown study of p21 to determine the relationship of the senescence growth arrest to SASP in beta cells. We found that Brd4 interacted with the many different nuclear proteins involved in transcription, including the inositol requiring enzyme 80 (Ino80) chromatin remodeling complex. We characterized the expression of Ino80 in NOD mice and human single-cell RNA-seq datasets and found its expression pattern to be consistent with a potential involvement in SASP. However, we were unable to achieve robust knockdown of this gene in NIT-1 cells, precluding our ability to determine its functional role. We also performed a functional study using siRNA knockdown to deplete p21, the main nuclear cell cycle arrest factor consistently upregulated in beta cell senescence in T1D and in ex vivo human islet culture models of senescence [8,11,16]. Surprisingly, we found that p21 is dispensable for maintenance of viability and for SASP gene activation in NIT-1 cells. Taken together, these data provide new insights about the regulation of SASP in beta cells.

## 2. Results

### 2.1. Identification of Brd4-Associated Proteins in the NIT-1 Beta Cell Line Senescence Model

**Figure 1:**
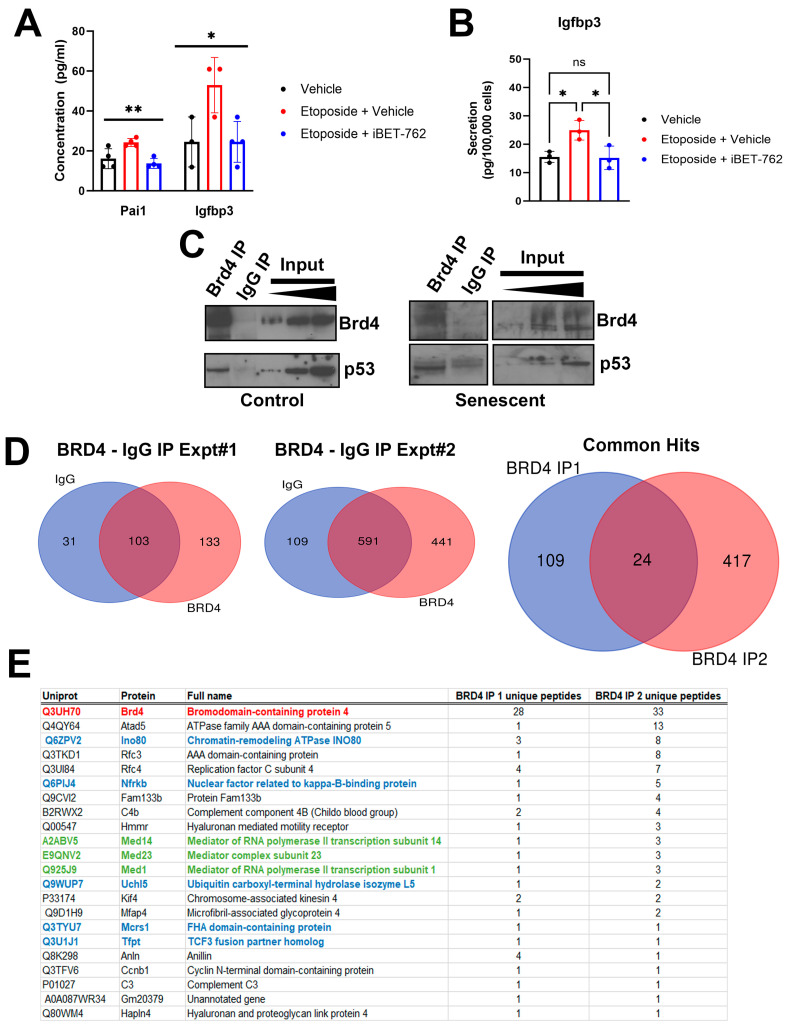
Regulation of SASP by BET proteins and identification of Brd4-associated proteins during senescence in NIT-1 cells. (**A**) Luminex assays measuring the concentration of SASP factors Pai1 and Igfbp3 in the 24 h conditioned media collected from NIT-1 cells control (vehicle, DMSO), or senescent cells (0.25 µM etoposide treatment) at 11 days post-etoposide washout. Following drug washout, cells were treated once with vehicle or iBET-762 (5 µM) until harvest. (**B**) A repeated experiment as in (**A**) except for assaying Igfpb3 secretion by normalizing the amount in the conditioned media to the viable cell counts at the end of the assay at day 7 post-etoposide washout. (**C**) Immunoprecipitation (IP) of Brd4 followed by Western blots for Brd4 and p53 from 100 µg of nuclear extract protein of control or senescent NIT-1 cells at day 6 post-etoposide treatment. Input is 3, 5, and 10 µg of nuclear extract and rabbit IgG was the species matched control IP. (**D**) Mass spectrometry was performed on IgG control and Brd4 IPs from senescent NIT-1 cell nuclear extracts in two independent experiments on senescent NIT-1 cells at day 6 post-etoposide treatment. The number of proteins identified uniquely or in common in the datasets are shown. Brd4 IP experiment 1 and 2 identified a total of 24 common accessions (22 different proteins). (**E**) Table listing the 22 different proteins in the Brd4 IP1 and IP2. Brd4 is highlighted in red, subunits of the Ino80 complex are in blue, and the Mediator complex is in green. ns, not significant, * *p* < 0.05, ** *p* < 0.005, one-way ANOVA.

To investigate the mechanisms of BET protein-mediated transcriptional activation of SASP, we used our previously established NIT-1 beta cell line model for senescence [16]. Etoposide treatment induces DNA double-strand break damage in these cells and leads to a cell cycle arrest, activation of the ataxia telangiectasia mutated (ATM)-p53–p21 DNA damage response pathway and a SASP, partially reflecting SASP in primary NOD mouse beta cells, involving Igfbp3 and Pai1 [16]. Senescence was induced using a sub-lethal dose of etoposide (0.25–0.5 µM) as previously, and following 72 h of culture, the media was replaced with fresh growth media lacking etoposide. The cells were then cultured for an additional 7–11 days to permit SASP activation. At day 6 or 10 post-etoposide, BET inhibitor iBET-762 was added to the media and the cells were cultured in serum-free media for 24 h to collect conditioned media (CM) for Luminex assay of SASP factors Igfbp3 and Pai1. This treatment leads to diminished expression of SASP genes and SASP factor secretion from primary NOD islet cells [11]. Senescent NIT-1 cells showed increased concentration of both factors in the CM relative to control NIT-1 cells treated with vehicle (DMSO), while the senescent cell group treated with iBET-762 showed decreased SASP factor concentrations resembling the control non-senescent cells (Figure 1A). The same result occurred when the assay was repeated and the secretion of Igfbp3 normalized to viable cell count (Figure 1B). These data suggest that NIT-1 cell SASP is dependent on BET proteins, as with primary NOD islet beta cells and human islets [11]. Thus, we determined that these cells can be used as a suitable model in which to study mechanisms of SASP activation by BET proteins.

BET protein Brd4 was previously found to bind to enhancer and promoter regions of SASP genes in islets of NOD mice [11]. To investigate how Brd4 drives SASP gene activation using the NIT-1 cell model, we performed immunoprecipitation–mass spectrometry (IP-MS) experiments to identify binding partners of Brd4 during senescence. Nuclear extracts were prepared from control or senescent NIT-1 cells at the 5 or 6 day post-etoposide removal timepoint, when previously we had shown that these cells are growth arrested and develop SASP [16], and we performed IP for Brd4. Notably, the Brd4 IP corresponded to ~6–10% of the total nuclear pool as judged from the input bands while the control IgG IP lacked Brd4, confirming specificity (Figure 1C). We also confirmed that the Brd4 IP contained a putative binding partner p53 [19] (Figure 1C), indicating that this antibody can capture protein complexes containing Brd4. Brd4 IP from both control and senescent NIT-1 nuclear extracts co-precipitated p53 (Figure 1C). We carried out IP-MS on nuclear protein extracts from senescent NIT-1 cells in parallel from Brd4 IP and control IgG IP in two independent experiments and cross-referenced the list of hits to identify proteins uniquely in the Brd4 IPs from both experiments (Figure 1D, Table S1). Through this approach, we identified 22 different proteins, including Brd4 as the top hit (Figure 1E). Among these proteins were those involved in transcription, DNA replication, and repair, including the previously reported DNA repair protein Atad5, subunits of the replication factor complex (Rfc3, Rfc4) [20], and subunits of the Mediator complex (Med14, Med23, Med1) [21]. Notably, we also identified several subunits of the Ino80 chromatin remodeling complex, including Ino80, Nfrkb, Uchl5, Mcrs1, and Tfpt [22,23] (Figure 1E), suggesting Brd4 associates with the Ino80 complex. Ino80 was previously demonstrated to interact with Brd4 as determined by two different large-scale high-throughput affinity purification studies in non-beta cell lines [21,24]. The Ino80 complex is a highly conserved ATP-dependent chromatin remodeling complex, involved in transcription, replication, and DNA repair [22] while Ino80 contributes to activation of super-enhancers in the context of cancer [25], a feature shared with Brd4 in the context of senescence [13]. Since Ino80 complex subunits were among the most highly represented hits in our two independent IP-MS experiments, we therefore focused on Ino80.

### 2.2. Characterization of Ino80 Expression in the Context of T1D

**Figure 2:**
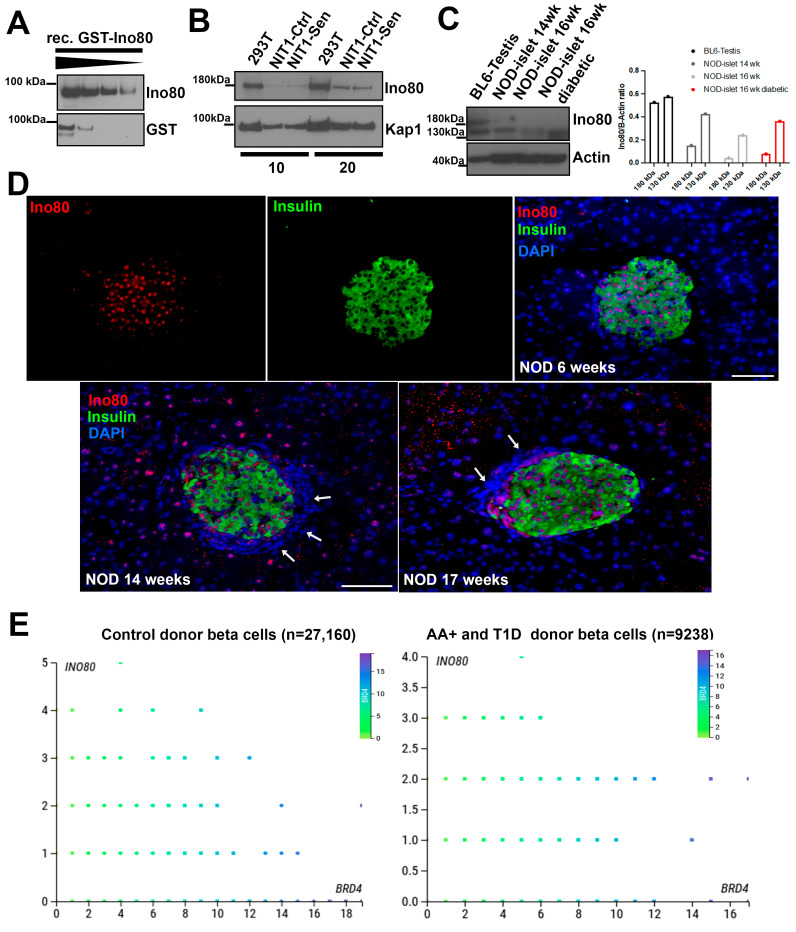
Characterization of Ino80 protein and *INO80* gene expression during T1D in NOD mice and humans, respectively. (**A**) Western blot analysis validating Ino80 antibody on a recombinant GST-tagged partial Ino80 protein (~90 kDa in size) with 200, 100, 50, 25 ng loaded per lane. (**B**) Western blot of Ino80 on 10 or 20 µg of nuclear extracts from human 293T cells as a control, or control and senescent NIT-1 cells at day 5 post-etoposide washout. Kap1 was a loading control. (**C**) Western blot of Ino80 on 10 µg of total RIPA protein extracts from 293T cells, C57BL6 3-month-old male testis and isolated islets from female NOD mice at indicated ages, including a 16-week-old diabetic NOD female. Beta-actin was a loading control. The 180 kDa and 130 kDa Ino80 bands from each sample were quantified relative to the beta-actin bands and plotted. (**D**) Immunohistochemistry and fluorescence staining of Ino80 along with insulin and DAPI as a nuclear counterstain on euglycemic NOD female mouse pancreas sections at 6 weeks, 14 weeks, and 17 weeks of age. Scale bars indicate 50 µm. (**E**) Analysis of publicly available single-cell RNA-seq data from PANC-DB (Human Pancreas Analysis Project) using CellxGene, comparing expression of *INO80* with *BRD4* across all beta cells in control donors (n = 27,160) or in autoantibody-positive (AA+) and T1D donors (n = 9238). Dots are colored according to *BRD4* expression levels. *Y*-axis reports binned expression level of *INO80* read-counts while the *X*-axis reports binned expression level of *BRD4* read-counts.

We characterized Ino80 expression by Western blotting on NIT-1 cells, NOD mouse islets, and using publicly available single-cell RNA-seq datasets from control, autoantibody-positive (AAB+) and T1D donors using the CellxGene platform on PANC-DB (Figure 2). First, we validated the specificity of a commercial antibody to Ino80 using a titration with GST-tagged recombinant Ino80 protein, which detected a single band at the size of the partial recombinant protein (~90 kDa) (Figure 2A). Next, we performed blotting of Ino80 on nuclear extracts from transformed human embryonic kidney cells (293T) alongside control and senescent NIT-1 cells. Ino80 protein was detected as a single band in nuclear extracts at ~180 kDa, and levels were similar between control and late-stage senescent NIT-1 cells during the timepoint when SASP is apparent (day 6 post-etoposide washout) (Figure 2B). Notably, we attempted to perform reciprocal IP of Ino80 to confirm interaction with Brd4, but were unable to successfully pull-down Ino80 using this antibody. We carried out blotting for Ino80 on whole cell extracts from primary cells, including C57BL6 mouse testis as a positive control [26] and isolated islets from female NOD mice (Figure 2C). Protein extract from testis showed a band at ~180 kDa as expected for full-length Ino80 with a lower molecular weight band at around 130 kDa also visible, potentially indicating a smaller isoform (Figure 2C). In samples of isolated islets from prediabetic female NOD mice at 14 weeks and in the samples at 16 weeks, (euglycemic or diabetic) only the smaller 130 kDa band was detected (Figure 2C) and quantification showed that while the 180 kDa band was decreased, the 130 kDa band intensity was similar between the 14-week-old, 16-week-old non-diabetic, and the 16-week-old diabetic NOD islets (Figure 2C). Given that islets from late stage and diabetic NOD mice have very few beta cells remaining, we reasoned that Ino80 expression may be predominantly in beta cells rather than immune cells. Therefore, we carried out immunofluorescence staining for Ino80 on pancreas sections from early stage (6 week) and late stage (14 and 17 week) euglycemic female NOD mice. This revealed prominent expression of Ino80 in the nucleus of Ins^+^ islet cells and some Ins^−^ islet cells, as well as in the nucleus of some acinar cells in the later stage mice (Figure 2D). No expression was found in the surrounding lymphocyte infiltrate (shown by the blue nuclei surrounding the Ins^+^ cells, white arrows, Figure 2D), suggesting that Ino80 expression is low in infiltrating immune cells.

To explore *INO80* expression in human beta cells, we surveyed publicly available single-cell RNAs-seq datasets generated by the Human Pancreas Analysis Program (HPAP) [27] on PANC-DB using the Cellxgene viewer (Figure 2E). We found that *INO80* was expressed in beta cells of control donors (n = 27,160 beta cells) as well as AA+ and T1D donors (n = 9238 beta cells in total), with no substantial differences in the distributions of gene expression levels between these groups (Figure 2E). AAB+ and T1D donors were combined for this analysis as the T1D donors only had 715 total beta cells. To examine whether *INO80* expression was correlated with *BRD4* in beta cells we generated scatterplots. These showed that *INO80* was not correlated with *BRD4* in control or AAB+ and T1D donors (Figure 2E). Together these data show that Ino80 protein is expressed in NOD mouse and the *INO80* gene is expressed in human beta cells in the context of T1D.

### 2.3. RNAi Knockdown to Investigate the Role of Ino80 and p21 during SASP in NIT-1 Cells

**Figure 3:**
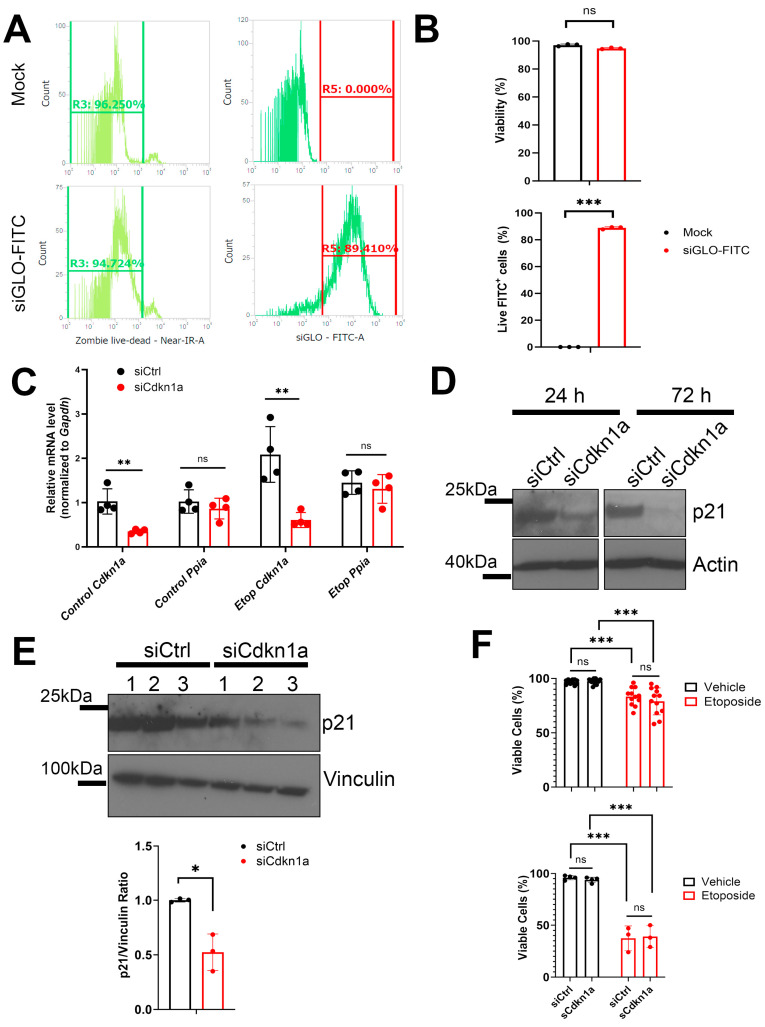
RNAi knockdown of *Cdkn1a* during senescence in NIT-1 cells. (**A**,**B**) Viability (gated R3) and transfection efficiency (gated R5) histograms and quantifications of NIT-1 cells transfected with 25 nM siGLO-FITC or mock transfected, 24 h post-transfection. (**C**) qRT-PCR analysis of *Cdkn1a* or *Ppia* as a control gene in control or etoposide induced senescent NIT-1 cells (72 h post-etoposide treatment) at 24 h post-transfection with control non-targeting siRNA (siCtrl), or *Cdkn1a* siRNA. N = 3 or 4 biological replicates per KD, error bars are S.D. ns = not significant, ** *p* < 0.005, one-way ANOVAs. (**D**) Western blot analysis of p21 on whole cell protein extracts prepared from NIT-1 cells transfected with the indicated siRNAs at 24 h or 72 h post-transfection. Beta-actin was as a loading control. (**E**) Western blot analysis of p21 levels after transfection with control or *Cdkn1a* siRNA at 72 h post-transfection in control/non-senescent NIT-1 cells in n = 3 biological replicates per transfection. Vinculin was a loading control. Plot shows quantification of p21 normalized to vinculin, error bars are S.D. * *p* < 0.05 two-tailed *t*-test. (**F**) Upper plot: viability assay using trypan blue staining and automated cell counting (Bio-Rad TC-20) of control or senescent NIT-1 cells transfected with the indicated siRNAs at 24 h post-transfection. Data are n = 10–12 biological replicates (upper plot), error bars are S.D. *Lower plot*: viability assay as in upper plot except 48 h following a second transfection which was conducted 2 days after the first transfection in control or senescent NIT-1 cells. Data are n = 3 or 4 biological replicates, error bars are S.D. ns = not significant, ** *p* < 0.005, *** *p* < 0.0005, two-tailed T-tests with multiple comparisons corrections (**C**,**E**) or two-way ANOVA in (**F**).

We next carried out functional studies to determine whether Ino80 is involved in regulating SASP in NIT-1 cells. For these studies, we chose to compare Ino80 with p21 (encoded by the *Cdkn1a* gene), which we previously showed is upregulated early following DNA damage and is sustained for 6–7 days post-damage in NIT-1 cells [16], likely as a mechanism mediating the growth arrest in these cells. p21 has also been shown to drive a unique secretome during senescence in other cell types [28]. We initially attempted to use a CRISPR-Cas9-based approach for generating constitutive population-level knockout (KO) of *Ino80* or *Cdkn1a* in NIT-1 cells using a commercially available kit (Synthego). However, we found that in a mixed population containing edited KO and non-edited wild-type cells, the non-edited wild-type cells out-proliferated the edited KO cells, such that they were progressively lost. Therefore, we turned to an RNAi-based approach using siRNAs to transiently knockdown (KD) *Ino80* or *Cdkn1a* in control and senescent NIT-1 cells (Figure 3). We first validated efficient transfection of siRNAs using a fluorescent reporter assay, which confirmed ~89% transfection efficiency with no loss of viability in comparison to mock cells (Figure 3A,B). Next, we transfected siRNAs for *Cdkn1a*, *Ino80*, or a control non-targeting siRNA into control or senescent NIT-1 cells, where the cells were first rendered senescent by etoposide treatment and after 72 h of etoposide transfected and assayed 24 h later (Figure 3C). Transfection of siRNAs against *Cdkn1a* showed ~60–70% KD of *Cdkn1a* mRNA relative to control siRNA (Figure 3C) while the *Ino80* siRNAs were less efficient, yielding only a ~20% in control conditions or a ~40% depletion in senescence from non-targeting control siRNA cells at 24 h post-transfection, which were not significant (Figure S1A). The housekeeping control gene *Ppia* was unaffected in both KD in control and senescent conditions (Figure 3C). Since the efficiency of *Ino80* KD was less than 50% at the mRNA level, we monitored the protein levels of each target following KD in NIT-1 cells under control conditions. p21 levels were found to decrease at 24 and 72 h post transfection (Figure 3D), consistent with the effect observed at the mRNA level at 24 h (Figure 3C). At 72 h, the *Cdkn1a* KD resulted in a significant 50–60% depletion of protein (Figure 3E). In contrast, *Ino80* was not depleted at 24 h at the protein level in control/non-senescent NIT-1 cells (Figure S1B). Due to the lack of efficiency in the *Ino80* KD both at the mRNA and the protein level, we could not further interrogate its role in SASP in NIT-1 cells.

Since interfering with DNA repair during the DNA damage response can trigger apoptosis [29] and p21 has been linked to efficient DNA repair and recovery from damage [22,30], we next measured viability of the p21 KD cells at the 24 h post-transfection timepoint using trypan blue staining. KD of *Cdkn1a* did not significantly affect NIT-1 cell viability at the 24 h post-transfection timepoint in control or senescent conditions, although reduced viability was apparent under senescent conditions compared with control (Figure 3F), consistent with our previous work [16]. To overcome limitations with the transient nature of the KDs and timing of monitoring later stage senescence phenotypes, we transfected control or senescent NIT-1 cells twice, once at the etoposide washout timepoint after 72 h of treatment and then again 2 days later and harvested cells 48 h after this second transfection (Figure 3F). We noticed a profound loss of viability 48 h following the second transfection in senescent NIT-1 cells when either control or *Cdkn1a* siRNA was used (Figure 3F), indicating that at later stages of senescence these cells are not permissive for transfection with this approach. Nevertheless, viability in the *Cdkn1a* KDs were not compromised relative to the control KD following a second transfeciton in the control cells (Figure 3F). Together these results indicate that while *Cdkn1a* is upregulated early in senescence induction in NIT-1 cells, wild-type levels are not required for maintenance of viability during early senescence or control conditions.

### 2.4. Effects of p21 Knockdown on SASP Gene Expression in NIT-1 Cells

**Figure 4:**
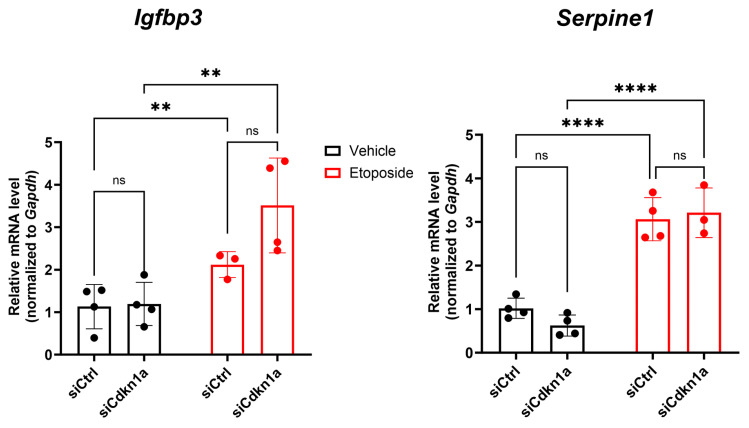
SASP gene activation is unaffected by KD of *Cdkn1a* in NIT-1 cells Control or senescent NIT-1 cells were transfected with indicated siRNAs and harvested 24 h post-transfection for qRT-PCR analysis of SASP genes *Igfbp3* and *Serpine1* (encoding Pai1). Data are n = 3 or 4 biological replicates, error bars are S.D. ns = not significant, ** *p* < 0.005, **** *p* < 0.0001, two-way ANOVA.

We next investigated the impact of *Cdkn1a* KD on SASP gene expression in NIT-1 cells. We monitored expression of SASP genes *Igfbp3* and *Serpine1* (which encodes Pai1) at the 24 h post-transfection timepoint where we established that the KD cells maintain viability under both control and senescent conditions and there is a consistent depletion of p21 at the protein level (Figure 3D). Both *Igfbp3* and *Serpine1* expression were increased in senescent NIT-1 cells as compared with the control (Figure 4), consistent with SASP gene activation and the observed increased secretion (Figure 1A,B). However, KD of *Cdkn1a* did not affect the induction of either *Igfbp3* or *Serpine1* during control or senescent conditions (Figure 4). Taken together, these data suggest that p21 is dispensable for SASP gene activation in NIT-1 cells.

## 3. Discussion

The SASP produced by senescent islets/beta cells from NOD mice and human islets ex vivo is regulated by BET proteins [8,11], but how BET proteins activate SASP genes in beta cells remains unclear. Here, we used our previously characterized NOD mouse-based beta cell line model for senescence [16] and noted that the SASP is similarly regulated by BET proteins, making these cells a suitable model to study molecular mechanisms of SASP. We found several previously identified Brd4-interacting proteins in senescent NIT-1 cells, including p53 [19] and subunits of the Ino80 complex [21,24]. Ino80 has diverse functions in DNA replication, recombination, transcriptional regulation, and DNA repair [22] and was previously found to interact with Brd4 in other cell types; thus, we chose to explore its role in SASP. Ino80 protein was expressed in the nucleus of primary beta cells in NOD mice and scRNA-seq data showed that the *INO80* gene is expressed in human beta cells from control, AA+, and T1D donors. These observations supported a potential role for Ino80/INO80 in regulating SASP in mouse and human beta cells. However, we were unable to generate stable CRISPR-edited *Ino80* KO NIT-1 cells and due to lack of efficient KD, we were unable to perform functional studies in NIT-1 cells to examine its role in SASP. Future studies should use a lentiviral delivered shRNA or inducible CRISPRi system to explore the role of this factor in NIT-1 cells.

We also investigated SASP regulation by performing functional studies on p21, the main cell cycle arrest factor during senescence induction in NIT-1 cells. KD of *Cdkn1a* (encoding p21) did not affect control or senescent NIT-1 cell viability or SASP gene activation. This was unexpected because p21 has been shown to drive a unique secretome in mouse fibroblasts, termed the “p21-associated secretory phenotype” (PASP) [28]. Beta cell specific deletion of the UPR regulator *Atf6*α in NOD mice leads to p21 upregulation and a PASP that mediates macrophage recruitment to islets [31]. This same study also showed that inhibiting p21 expression reduced expression and secretion of PASP factors from beta cells [31]. Thus, we expected p21 depletion to compromise SASP activation in NIT-1 cells. However, studies in other cell types have shown that the cell cycle arrest during DNA damage induced senescence enforced by p21 or p16^Ink4a^ can be decoupled from a canonical inflammatory SASP [32]. Indeed, in primary human IMR-90 and WI-38 lung fibroblasts, SASP is dependent on the DNA damage response involving ATM signaling and chromatin remodeling [33], but does not require p53 [34]. This suggests that during senescence induction by DNA damage in NIT-1 cells, upregulation of p21 occurs independently of SASP activation. Although we did not explore this mechanism in human islets and we found *INO80* is expressed in human beta cells, we found no clear relationship between *INO80* and *BRD4* expression in control versus AAB+ and T1D donors (Figure 2E). Our previous studies modeling senescence induction using human islets are consistent, since we showed that BET protein inhibition diminishes *CDKN1A* expression from human islets following senescence induction ex vivo [11]. The finding that Brd4 interacted with p53 in control and senescent NIT-1 cells (Figure 1C) supports the idea that BET proteins may promote p53-dependent activation of *Cdkn1a/CDKN1A* during senescence in beta cells. Additional experiments are required to test this hypothesis.

In addition, to an effect on SASP, we also expected depletion of p21 to acutely reduce NIT-1 cell viability under early senescence conditions. p21 has been linked to DNA repair [30] and was found to promote a pro-survival phenotype in other cell lines during senescence [35]. p21 was also reported to control MIN6 beta cell viability acutely following DNA damage [36] and reducing p21 levels led to increased apoptosis in primary NOD islet cells [31]. However, in both of these studies [31,36], p21 expression was inhibited using the chemical inhibitor UC2288, which inhibits *Cdkn1a* expression transcriptionally or post-transcriptionally independent of p53 leading to reduced protein [37]. It is unclear whether this inhibitor has any other effects that could explain an impact on beta cell viability. The original paper reporting this compound showed that it reduced cell viability in several cancer cell lines by ~20% independently of an effect on p21 levels [37]. UC2288 also did not affect p21 expression uniformly throughout the cell; an effect was observed in reducing cytosolic but not nuclear p21 [37] and the studies using UC2288 on beta cells did not investigate whether it impacts nuclear or cytosolic p21. A side-by-side comparison of genetic and chemical-based approaches would be required to establish whether UC2288 has any off-target or cytotoxic effects in beta cells beyond its effect on p21 levels.

## 4. Conclusions

In conclusion, we found a list of candidate Brd4-interacting proteins, some of which could participate in SASP regulation in NIT-1 cells. We found that subunits of the Ino80 chromatin remodeling complex were associated with Brd4 in senescent NIT-1 cells. However, we were unable to validate this interaction using an orthogonal approach, and so further studies should be carried out to corroborate the IP-MS studies performed. We characterized the expression of Ino80 at the protein and gene level during the progression of T1D in NOD mice and humans. We further showed that normal levels of p21 are not required for maintaining viability following DNA damage or SASP gene activation in the NIT-1 beta cell model of senescence. This could suggest that p21 is dispensable for the SASP during beta cell senescence and therefore could represent a distinct molecular target that controls only the cell cycle arrest. Further investigations will be required to explore this in more clinically and physiologically relevant models, such as primary human islets. A major caveat and limitation of our studies is that the siRNA KD was transient, and we could not achieve stable CRIPSR-edited KO of *Cdkn1a* in NIT-1 cells. It is possible that phenotypes would occur using approaches for stably disrupting *Cdkn1a* expression and an inducible KO or KD system using clonally derived cells would be required to evaluate these possibilities. Regardless, our results provide insights into the molecular control of SASP in beta cells in the context of T1D.

## 5. Materials and Methods

### 5.1. Cell Culture and RNAi

NOD mouse-derived beta cell line NIT-1 [17] was cultured as previously [16] in DMEM high glucose containing 10% fetal bovine serum (FBS, Millipore-Sigma, Burlington, VT, USA), 1 mM sodium pyruvate, 1X antibiotic-antimycotic (Gibco), and 50 µM mercaptoethanol. Cells were seeded at 2–3 *×* 10^6^ cells per 75 cm^2^ flask and split every 5–7 days. The 293T cells were cultured in DMEM high glucose containing 10% FBS and 1X antibiotic–antimycotic (ThermoFisher, Waltham, MA, USA). Cells were confirmed mycoplasma-free using a commercial kit (HEK-Blue2 indicator cells, Invivogen, San Diego, CA, USA). Senescence-like growth arrest was induced in NIT-1 cells as previously [16], by treating cells with 0.25–0.5 µM etoposide (Millipore-Sigma) for 72 h, followed by replacing the media with drug-free media and culturing cells for an additional 1–7 days, depending on the assay. RNAi knockdown was achieved using siRNA SMARTpools of 4 independent siRNAs targeting the gene of interest (Horizon Discovery, Cambridge, UK) or a non-targeting siRNA (non-targeted siRNA pool #2). Transfection efficiency was confirmed using siGLO-FITC reagent (Horizon Discovery) and optimal cationic carrier reagent (DharmaFECT1) complexed in Opti-Mem serum free media (ThermoFisher) with 25–50 nM of each siRNA. NIT-1 cells were seeded at 3–4 × 10^5^ cells/well for etoposide treatment or 2 × 10^5^ cells/well for vehicle control treatment in 12-well plates, and senescence induction or vehicle treatment was performed as indicated above, and at the 72 h post-etoposide timepoint, the etoposide was removed and cells were incubated with 25–50 nM of each siRNA complexed with DharmaFECT1 reagent (Horizon Discovery) in fresh antibiotic-free growth media for 24 h. Following this incubation, cells were harvested, counted using trypan blue staining and used for other assays. For long-term storage and RNA or protein extraction, cell pellets were frozen at −80 °C.

### 5.2. Immunopreciptation and Mass Spectrometry

Nuclear extracts were prepared from NIT-1 cells on day 5 or 6 post-etoposide washout after the cells had arrested proliferation, using the high-salt extraction method according to previous studies [38]. Nuclear protein was quantified using BCA assay (ThermoFisher). For Western blotting, 100 μg of nuclear extract from control or senescent NIT-1 cells was incubated with 1–2 µg of rabbit IgG or anti-BRD4 antibodies (Bethyl/Fortis Life Sciences, Waltham, MA, USA, cat # A301–985A100) for 1 h at 4 °C with rotation, according to the product instructions. Beads were immobilized on a magnetic rack stand and washed 4 times in IP wash buffer (ThermoFisher). Samples were eluted by heating at 70 °C in SDS-PAGE loading buffer (1X LDS sample buffer, ThermoFisher), again magnetized, and eluates were run immediately on SDS-PAGE gels or stored at −20 °C. For mass spectrometry, 500 μg of nuclear extract from control or senescent NIT-1 cells was incubated with 3 µg of rabbit IgG or anti-BRD4 antibodies covalently linked to protein G-dynabeads (ThermoFisher) for 1 h at 4 °C with rotation, according to the product instructions. Beads were immobilized on a magnetic rack stand and washed 5 times in IP wash buffer (ThermoFisher) and eluted by heating in SDS-PAGE loading buffer (1X LDS sample buffer, ThermoFisher). Immunoprecipitated samples along with nuclear extract inputs were visualized by colloidal Coomassie blue G-250 staining using GelCode Blue (ThermoFisher). For mass spectrometry, beads were submitted to the Manitoba Centre for Proteomics and Systems Biology for on-bead trypsin digestion and elution for liquid chromatography tandem mass spectrometry (LC-MS/MS) on an ObiTrap instrument (ThermoFisher). MS spectra were matched against the mouse UniProt database. Hits were considered proteins with >2 unique peptides. Complete lists of proteins identified in all IP-MS samples are shown in Table S1, while a list of the top 22 proteins only recovered in the Brd4 IP from two independent experiments is shown in Figure 1E.

### 5.3. Western Blotting

Western blot analysis was performed as previously described [16]. Approximately 30–40 isolated islets pooled from 14-week-old nondiabetic, 16-week-old nondiabetic or 16-week-old diabetic (>15 mM two blood glucose measurements) female mice were kindly provided by Dr. Anil Bhushan (UC San Francisco). Testis tissue from 4-month-old male C57BL6 mice was kindly provided by Dr. Christine Doucette (University of Manitoba). Tissue collection from research animals was conducted in accordance with local institutional animal care ethics guidelines at each institution. Whole cell protein extracts were prepared using RIPA buffer containing 1X Halt protease–phosphatase inhibitor cocktail (ThermoFisher). Tissue or islets were dissolved by vortexing in RIPA buffer, incubated on ice for 15 min and clarified by centrifugation at 14,000× *g* for 5 min. Tissue or islet extracts and nuclear extracts were quantified by BCA assay (ThermoFisher). Purified recombinant Ino80 protein (Proteintech, Rosemont, IL, USA, cat #Ag20309), IP samples, nuclear extracts from NIT-1 cells or 293T cells, or total radio immunoprecipitation assay (RIPA) buffer extracted protein from islets or testis were heated for 10 min at 70 °C in SDS-PAGE loading buffer (1X LDS sample buffer, ThermoFisher), run on 4–12% gradient SDS-PAGE gels (Bolt-Bis Tris Plus, ThermoFisher) and transferred to nitrocellulose membranes using an iBlot2 apparatus according to the default P0 program. Membranes were blocked in 5% non-fat milk in Tris-buffered saline (TBS), pH 7.6 for 1 h at room temp and probed with primary antibodies including 1:500 anti-p21 (Santa Cruz sc-6246), 1:1000 anti-Ino80 (Proteintech, cat# 18810-1-AP), 1:5000 anti-beta-actin (Cell Signaling Technology, Danvers, MA, USA, cat#4970), 1:5000 anti-KAP1 (Novus Biologicals, Toronto, ON, Canada, NB100-74549) or anti-vinculin (1:1000, Cell Signaling Technology, cat# 13901) all diluted in TBS containing 0.1% Tween-20 (TBS-T) at 4 °C for 18–24 h. Membranes were washed 3 times in TBS-T and probed with HRP-conjugated secondary antibodies for 1 h at room temp followed by ECL detection (WestPico Plus, ThermoFisher) and X-ray film exposure. Several exposures were captured for each membrane to ensure signals were within linear ranges. To quantify loading controls on the same blots, membranes were stripped with Restore Plus Stripping Buffer (ThermoFisher) for 15 min at room temperature, blocked again for 1 h in 5% milk with TBS-T and probed for loading controls. Quantification of band intensities were performed using ImageJ software and normalized to loading control proteins.

### 5.4. Immunohistochemistry

Immunohistochemistry was performed as previously described [18]. Formalin fixed paraffin embedded pancreas tissue sections from 6-, 14-, and 17-week-old euglycemic NOD/ShiLtJ female mice (gift from A. Bhushan, UCSF) were rehydrated with xylene–ethanol washes, and antigen retrieval was performed with citrate buffer pH 6.0 (Vector Labs, Newark, NJ, USA) in a lab-grade pressure cooker (BioSB Tinto Retrieval system). Tissue collection from research animals was conducted in accordance with local institutional animal care ethics guidelines at UCSF. Tissue sections were blocked with 2% normal donkey serum (Millipore-Sigma) in protein blocker buffer (Abcam, Cambridge, UK) for 15 min and probed with 1:250 rabbit anti-Ino80 (Proteintech cat# 18810-AP-1) and 1:2 guinea pig anti-insulin (Agilent Technologies, Santa Clara, CA, USA, cat #IR002) in antibody diluent (Agilent Technologies) for 24 h at 4 °C. Slides were washed in TBS-T and probed at 1:200 dilution in antibody diluent with appropriate secondary antibodies raised in donkey: anti-rabbit IgG Alexa594 and anti-guinea pig Alexa 488, respectively. Sections were counterstained and mounted with Pro-Long Diamond anti-fade mountant with DAPI (ThermoFisher) and imaged on a Zeiss Axioscope 2 epifluorescence microscope.

### 5.5. Quantitative Reverse Transcriptase PCR (qRT-PCR)

qRT-PCR was performed using the SYBR green method as previously described [16]. NIT-1 total RNA was extracted and DNase I treated using the Direct-zol total RNA microprep kit (Zymogen). Approximately 100 ng of total RNA was used to synthesize cDNA using the LUNA RT kit (New England Biolabs, Ipswich, MA, USA). qPCR was performed using the LUNA Universal Master Mix (New England Biolabs) on a Bio-Rad CFX96 instrument. Primer sequences used here are shown in Table S2. Gapdh was used as a housekeeping gene. Fold-changes were calculated using the delta–delta C_t_ method.

### 5.6. Single Cell RNA-Seq Data Analysis

Human pancreas single-cell RNA-seq datasets were publicly available through the Human Pancreas Analysis Program PANC-DB resource https://hpap.pmacs.upenn.edu/analysis [27,39], accessed on 20 December 2023. We used the interactive browser tool Cellxgene viewer to view expression of INO80 and BRD4 across all beta cells in control donors (n = 27,160 beta cells) versus AAB+ and T1D donors (n = 9238 beta cells). AAB+ and T1D donor beta cells were combined due to the low number of beta cells in the T1D donors (only 715 cells). Expression data were binned by heat-color map on BRD4 expression level and expression of INO80 was plotted relative to BRD4 expression. Screenshots were captured of the scatterplot datasets for Figure 2E.

### 5.7. Flow Cytometry

NIT-1 cells transfected with siGLO-FITC (Horizon Discovery) or mock transfected were trypsinized, washed in PBS, and counted. Cells were incubated at 1 × 10^6^ cells/mL with 1:1000 dilution of Zombie near IR fixable viability dye (BioLegend, San Diego, CA, USA) for 30 min on ice in cell staining buffer (BioLegend). Cells were washed by adding 400 µL of cell staining buffer, and centrifuging at 300× *g* for 5 min. Cells were then analyzed by flow cytometry on an Attune acoustic focusing cytometer equipped with 488 nm and 633 nm lasers. Gating was as shown in Figure 3A: FSC-A/SSC-A (cells), FSC-A/FSC-W (singlets), Zombie Near IR-negative (live cells), siGLO-FITC-positive. Percent siGLO positive cells were determined relative to the mock transfected cells, which were set to ~0% FITC-positive.

### 5.8. Luminex Assays

Luminex multi-analyte bead assays were performed as previously described [16]. Commercial custom panels (R&D Systems, Minneapolis, MN, USA) were designed to assay secreted Igfbp3 and Pai1. Conditioned media were collected at indicated time-points post-etoposide treatment, clarified by centrifugation at 3000× *g* for 5 min and stored at 4 °C until assays were carried out. Data were reported as concentration (pg/mL) or normalized to cell count (pg/100,000 cells).

### 5.9. Statistics

All quantitative experiments were performed with a minimum of n = 3 or 4 biological replicates and all error bars are S.D. Two group comparisons were performed with unpaired two-tailed *t*-tests, while comparisons of three or multiple groups across two variables were performed using one- or two-way ANOVA where indicated. Multiple comparison corrections were done using Tukey test. Statistical testing and significance were calculated on GraphPad Prism version 10.1.2 and results considered significant at *p* < 0.05.

## Data Availability

Publicly available scRNA-seq datasets from control, AAB+, and T1D donors were generated via PANC-DB CellxGene viewer.

## Supplementary Materials

The following supporting information can be downloaded at: https://www.mdpi.com/article/10.3390/epigenomes8010010/s1, Table S1: Proteins identified by Mass Spectrometry in Brd4 and IgG IP. Table S2: List of primers used for qPCR in this study. Figure S1: RNAi knockdown of Ino80 in NIT-1 cells. Control or senescent NIT-1 cells were transfected with control or Ino80 siRNAs at 72 h post-etoposide or vehicle (DMSO) treatment. Cells were harvested 24 h after transfection for analysis of Ino80 mRNA by qRT-PCR (A) or Ino80 protein by western blot (B). Cells transfected with Ino80 siRNAs did not show significant reduction of Ino80 mRNA or protein levels. Data are means and error bars are S.D. ns = not significant by two-tailed T-tests.
