# Supplementary material for: Exploring Transcriptional Regulation of Beta Cell SASP by Brd4-Associated Proteins and Cell Cycle Control Protein p21
Source: Epigenomes. 2024 Mar 6;8(1):10. doi: 10.3390/epigenomes8010010 (PMC10968907; doi:10.3390/epigenomes8010010)
Supplement: Supplementary file 1 [file epigenomes-08-00010-s001.zip › Figure S1.pdf]

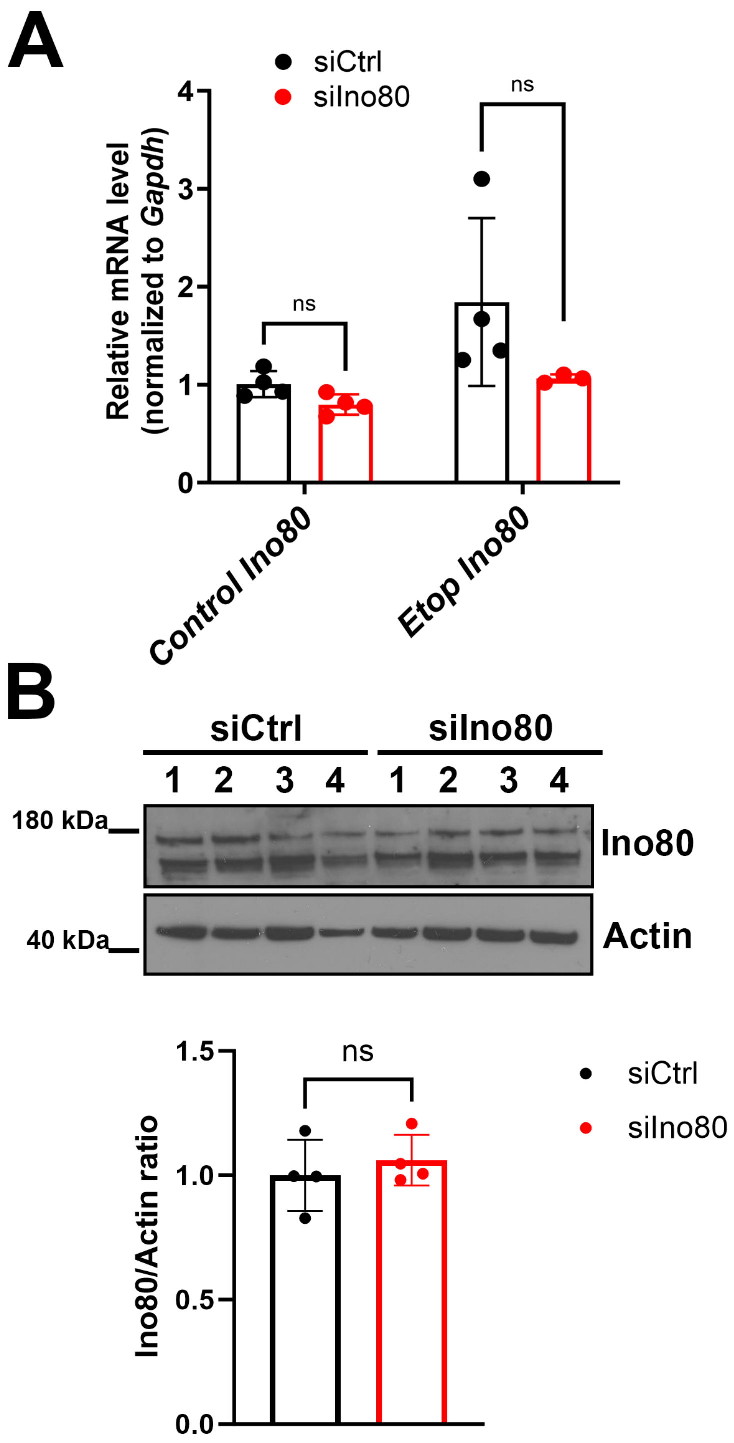

Figure S1. RNAi knockdown of *Ino80* in NIT-1 cells. Control or senescent NIT-1 cells were transfected with control or Ino80 siRNAs at 72 h post-etoposide or vehicle (DMSO) treatment. Cells were harvested 24 h after transfection for analysis of *Ino80* mRNA by qRT-PCR (A) or Ino80 protein by western blot (B). Cells transfected with Ino80 siRNAs did not show significant reduction of *Ino80* mRNA or protein levels. Data are means and error bars are S.D. ns = not significant by two-tailed T-tests.
